# Subthalamic Deep Brain Stimulation Affects Plasma Corticosterone Concentration and Peripheral Immunity Changes in Rat Model of Parkinson’s Disease

**DOI:** 10.1007/s11481-020-09934-7

**Published:** 2020-07-09

**Authors:** Beata Grembecka, Wojciech Glac, Magdalena Listowska, Grażyna Jerzemowska, Karolina Plucińska, Irena Majkutewicz, Piotr Badtke, Danuta Wrona

**Affiliations:** 1 Department of Animal and Human Physiology, Faculty of Biology, University of Gdańsk, 59 Wita Stwosza Str, 80-308 Gdańsk, Poland; 2 Department of Physiology, Medical University of Gdańsk, 1 Dębinki Str, 80-211 Gdańsk, Poland

**Keywords:** Deep brain stimulation of subthalamic nucleus, 6-hydroxydopamine rat model of Parkinson’s disease, Corticosterone, Lymphocytes, Cytokines, Apoptosis

## Abstract

Deep brain stimulation of the subthalamic nucleus (DBS-STN) is an effective treatment for advanced motor symptoms of Parkinson’s disease (PD). Recently, a connection between the limbic part of the STN and side effects of DBS-STN has been increasingly recognized. Animal studies have shown that DBS-STN influences behavior and provokes neurochemical changes in regions of the limbic system. Some of these regions, which are activated during DBS-STN, are involved in neuroimmunomodulation. The therapeutic effects of DBS-STN in PD treatment are clear, but the influence of DBS-STN on peripheral immunity has not been reported so far. In this study, we examined the effects of unilateral DBS-STN applied in male Wistar rats with 6-hydroxydopamine PD model (DBS-6OHDA) and rats without nigral dopamine depletion (DBS) on corticosterone (CORT) plasma concentration, blood natural killer cell cytotoxicity (NKCC), leukocyte numbers, lymphocyte population and apoptosis numbers, plasma interferon gamma (IFN-γ), interleukin 6 (IL-6), and tumor necrosis factor (TNF-α) concentration. The same peripheral immune parameters we measured also in non-stimulated rats with PD model (6OHDA). We observed peripheral immunity changes related to PD model. The NKCC and percentage of T cytotoxic lymphocytes were enhanced, while the level of lymphocyte apoptosis was down regulated in 6OHDA and DBS-6OHDA groups. After DBS-STN (DBS-6OHDA and DBS groups), the plasma CORT and TNF-α were elevated, the number of NK cells and percentage of apoptosis were increased, while the number of B lymphocytes was decreased. We also found, changes in plasma IFN-γ and IL-6 levels in all the groups. These results suggest potential peripheral immunomodulative effects of DBS-STN in the rat model of PD. However, further studies are necessary to explain these findings and their clinical implication.

**Graphical Abstract:**
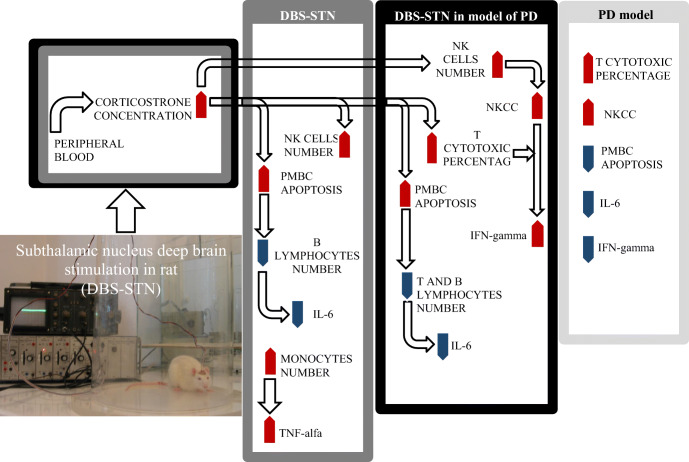
Influence of deep brain stimulation of the subthalamic nucleus on peripheral immunity in rat model of Parkinson’s disease.

Influence of deep brain stimulation of the subthalamic nucleus on peripheral immunity in rat model of Parkinson’s disease.

## Introduction

Numerous structural and functional connectivity studies have indicated that the subthalamic nucleus (STN) is an important point of integration of both motor and associative/limbic inputs into the basal ganglia circuit (Haegelen et al. 2009; Baunez et al. 2011). This observation confirms motor and non-motor effects of deep brain subthalamic nucleus stimulation (DBS-STN) used for the treatment of Parkinson’s disease (PD) patients (Nassery et al. 2016), and seen in animal model data (Baunez et al. 2011). DBS-STN alleviates motor complications and allows drastic reductions in medication in Parkinsonian patients (Benabid et al. 2009). However, the beneficial effects of DBS-STN treatment in some patients often correlate with the onset of psychiatric symptoms, as reported in the literature (Castrioto et al. 2014). The most common psychiatric effects reported after DBS-STN are depression, apathy, emotional reactivity, and hypomania (Nassery et al. 2016). Animal studies have shown that DBS-STN provokes neurochemical changes in limbic associative regions (Winter et al. 2008; Aleksandrova et al. 2013). Some of the limbic regions activated during DBS-STN in rats are involved in neuroimmunomodulation (Wrona 2006).

DBS-STN influences the hypothalamic-pituitary-adrenal (HPA) axis activity in PD patients (Ružička et al. 2015) and changes corticosterone (CORT) secretion (Ružička et al. 2012). In addition, in rats the STN is connected with hypothalamic nuclei (Cavdar et al. 2018). It is well known that glucocorticoids and cytokines are involved in several responses triggered by a variety of environmental and physiological stimuli. In the last decade, several studies have supported the hypothesis whereby the innate immune system and inflammation drive the neurodegenerative processes linked with PD symptoms (Hirsch et al. 2012; Fuzzati-Armentero et al. 2019; Kustrimovic et al. 2019; Tansey and Romero-Ramos 2019). Additionally, PD is associated with changes in secretion of stress-related hormones and cytokines (Reale et al. 2009; Fuzzati-Armentero et al. 2019). These changes support a role for inflammatory response in initiating and sustaining the central and peripheral immune mechanisms which trigger neurodegeneration. An animal study has confirmed that central dopamine depletion causes transient changes in blood leukocyte distribution and cytokine production (Engler et al. 2009).

While the therapeutic effects of DBS-STN in Parkinson’s disease treatment are clear, little is known about the influence of DBS-STN on immune system mechanisms, including natural killer cell cytotoxicity (NKCC), NKCC-related factors and lymphocyte populations in peripheral blood. In our previous study, we demonstrated that limbic regions such as the medial septum (MS) (Podlacha et al. 2016), bed nucleus of the stria terminalis (BST) (Myślińska et al. 2012) and ventral tegmental area (VTA) (Wrona et al. 2004) are involved in neuroimmunomodulation in rats. The neuroimaging and behavioral study confirms that the STN plays an important role in limbic function (Rossi and Gunduz 2015), also indicated by the side effects of DBS-STN in PD patients. Using this as a basis, in our current study we aimed at analyzing changes in peripheral endocrine and immune responses after DBS-STN in rats. Specifically, we investigated the impact of DBS-STN on plasma CORT concentrations and then analyzed immune parameters such as: the number and function of blood lymphocytes, peripheral blood mononuclear cells (PBMC) apoptosis and plasma cytokine concentration in rats.

## Materials and Methods

### Animals

Male Wistar rats (*n* = 49) (Tri-City Central Animal Laboratory, Research and Service Centre of the Medical University of Gdansk, Poland), weighing 280–300 g at the time of surgery, were used for the experiments. Animals were individually housed in standard plastic cages with elevated metal wire lids and were maintained on a 12:12-h light/dark cycle (lights on at 06.00 AM) with ad libitum access to standard rat diet and water. Rats were acclimatized to the new surroundings for 2 weeks before initiation of any experimental procedure. Additionally, animals were handled on a daily basis to minimize stress caused during experimental procedures. Animals were split into three experimental groups:

DBS group – DBS-STN without PD induction (*n* = 12),

DBS-6OHDA group – DBS-STN after intranigral (substantia nigra pars compacta, SNpc) injection of 6-hydroxydopamine (6-OHDA) to mimic PD (*n* = 14),

6OHDA group – non-stimulated rats with intranigral injection of 6-OHDA (*n* = 9).

SHAM – control group, electrode implantation in the STN with vehicle injection into SNpc (n = 14).

All the procedures were approved by the Local Ethical Committee for the Care and Use of Laboratory Animals of the Medical University of Gdansk, Poland and are in accordance with the EU Directive 2010/63/EU.

**Fig. 1:**
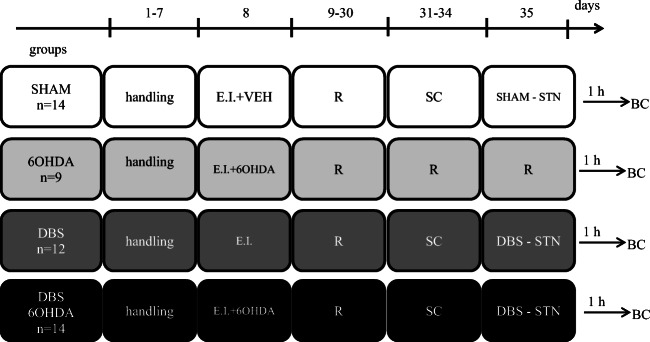
Diagram of experimental procedures and group assignments, Explanations: E.I. – implantation of a stimulating electrode into the STN, VEH – injection of vehicle into the SNpc, 6OHDA – injection of 6-hydroxydopamine into the SNpc, R – recovery period in the home cage, SC – screening procedure before sham or DBS-STN, SHAM – STN sham stimulation, DBS-STN – STN deep brain stimulation, BC –blood collection (1 h after the end of the procedure appropriate for the group)

Figure 1 shows a diagram of the experimental procedure.

### Brain Surgery

#### 6-OHDA Lesion

Animals were fully anaesthetized with pentobarbital anesthesia (50 mg/kg, i.p., Vetbutal, Biowet Pulawy, Poland) and atropine sulfate (0.25 mg/kg, s.c, Polfa, Poland) and placed in a stereotactic apparatus (Kopf Instruments, USA). The skull was exposed by a midline incision of the skin and a hole was drilled above the lesion site. The neurotoxin 6-OHDA (6-hydroxydopamine HCl, Sigma–Aldrich, Poland) was injected into the right SNpc in a volume of 4 μl (3 μg/μl dissolved in 0.9% NaCl containing 0.1% ascorbic acid). The following coordinates from the rat stereotactic atlas (Paxinos and Watson 2007) were used (in reference to bregma): anteroposterior (AP) – 5.3, lateral (L) – 2.4, dorsoventral (DV) – 7.5. Injections were performed using a microsyringe with a 26-gauge needle (Hamilton, USA) that was attached to a microinjection unit (Model 5000, Kopf Instruments, USA). The injection rate was 0.5 μl/min and the cannula was left in place for additional 5 min after injection to allow for diffusion into the tissue. To protect noradrenergic neurons from damage, animals received an intraperitoneal injection with the noradrenaline reuptake inhibitor desipramine (25 mg/kg, Sigma-Aldrich, Poland) 30 min prior to neurotoxin injection (Fulceri et al. 2006). Sham-lesioned controls underwent the same procedure but received vehicle instead of 6-OHDA.

#### STN Electrode Implantation

After 6-OHDA microinjection, animals were implanted unilaterally with a monopolar, stainless steel electrode (0.2 mm diameter, Plastic One, Germany) in the right STN. The stereotaxic coordinates from bregma (Paxinos and Watson 2007) were: AP − 3.6 mm; L − 2.6 mm; D − 8.0 mm. The electrode was anchored to four stainless steel skull screws with dental acrylic (Duracryl Plus, Spofa Denta, Czech Republic), a stainless steel wire soldered to a screw served as the anode for electrical stimulation. Rats postoperatively received antibiotic solution (Penicillin procaine, Polfa, Poland).

Rats were allowed to recover for 21 days after the brain surgery before being habituated to DBS-STN or sham procedure.

### Deep Brain Stimulation

After a three-week recovery period, rats were habituated to DBS-STN conditions for three consecutive days. Animals were taken from their home cages and placed in transparent plexiglas testing cylinders (gauge: 30 cm; height: 40 cm) and were allowed to explore the box for 30 min. Within the first 5 min of screening stimulation, the behavioral effects of increasing the stimulation intensity from 0 to 150 μA were examined in individual animals. During this procedure, contralateral torsion of the head or dyskinetic movements of the contralateral forelimb were observed. A range of current intensity was set at 30–125 μA at values which were just below the threshold for dyskinesias. The following day, that intensity of current was applied continuously for 1 h throughout the DBS-STN procedure. The stimulation duration and parameters were determined based on the method described by Salin et al. (2002). Stimuli were delivered by a stimulator unit (215/T, Hugo Sachs Elektronik D7806 March F.R., Germany) that gave rectangular pulses. The frequency was set at 130 Hz and the pulse width at 60 μs all over the stimulation period for all the stimulated animals. Sham-stimulated controls underwent the same procedure excluding the current flow.

### Blood Collection

Blood samples were collected by heart puncture (Narcotane anesthesia; Zentiva, Czech Republic) between 09.00 and 10.00 AM when CORT levels are at their lowest (Ulrich-Lai and Herman 2009) and 1 h after the electrical or sham stimulation. The blood samples were divided into two tubes. One of tube was centrifuged (10 min, 3000 g) to obtain fresh plasma without platelets and cells. The supernatant was transferred to Eppendorf tubes, quickly frozen at −70 °C and stored to analyze plasma CORT and cytokines: tumor necrosis factor (TNF-α), interferon gamma (IFN-γ), interleukin 6 (IL-6) concentrations. The second part of the sample was tested immediately for natural killer cell activity (NKCC), flow cytometric analysis of apoptosis and the number of T, B, NK, T helper and T cytotoxic lymphocyte subsets.

### Plasma CORT and Cytokine Determination

Plasma CORT concentrations were measured by radioimmunoassay using a commercially available kit (Rat corticosterone ^125^I RIA Kit, MP Biomedicals, USA) and Wizard 1470 gamma counter (Pharmacia-LKB, Finland). The minimal detectable dose in this system was 7.7 ng/ml.

Concentrations of cytokines in the plasma were quantified using an enzyme-linked immunoassay method (ELISA) with a commercially available kit (Rat TNF-α, Rat IFN-γ, Rat IL-6 ELISA Kits, Thermo Scientific) as previously described (Wrona et al. 2014; Podlacha et al. 2016). Samples were prepared according to the manufacturer’s instructions and were analyzed on DTX880 Multimode Detector (Beckman Coulter, USA) system set to 450 nm. The cytokine concentrations were calculated based on the standard curve. The sensitivity of detection was 16 pg/ml for IL-6, 15 pg/ml for TNF-α, and 2 pg/ml for IFN-γ.

### NKCC Assay

NKCC was quantified using a ^51^Cr release assay. The PBMC were used as the effector (E) cells against YAC-1 murine tissue culture cell line as target (T) as previously described in details (Wrona et al. 2014; Podlacha et al. 2016).

#### Preparation of PBMC – Effector Cells

For the determination of the NKCC, PBMC as E cells were separated from heparinized blood by the Ficoll 400 (Pharmacia, Uppsala Sweden) and Uropolinum (Polfa, Starogard, Poland) by density centrifugation method. After the centrifugation (1113 x g, 30 min at 4 °C) the isolated cells were collected with a Pasteur pipette, washed with phosphate – buffered saline (PBS) three times, counted and adjusted to 1 × 10^6^ cells/ml in complete medium.

#### Target Cells

The YAC–1 tissue culture cell line of YAC was used as target cells for determination of NKCC. The cells were maintained in a stationary suspension culture in complete medium: RPMI 1640 (Sigma – Aldrich, Poland), and penicillin (100 U/ml, Polfa, Tarchomin, Poland) in a 5% CO_2_ humidified incubator (standard culture conditions). Labelled with 100 μl of Na_2_^51^CrO_4_ (Radio Chemical Centre, Otwock – Świerk, Poland), the cells were washed five times using 2% RPMI 1640 (Sigma – Aldrich, Poland) and then adjusted to 1 × 10^5^/ml in complete medium with various concentrations of effector (E/T = 50:1, 25:1, 12:1, in a total volume of 200 μl) in triplicate under standard culture conditions for 4 h. The spontaneous ^51^Cr release wells had T cells plus 100 μl of complete medium, while the maximum release wells contained T cells plus 100 μl of complete medium with 5% Triton X – 100 (Sigma – Aldrich, Poland) and the experimental wells contained 100 μl of E cells plus 100 μl of T cells. After determination of the experimental (Exp), spontaneous (Sp) and maximal (Max) ^51^Cr release, the percent of specific lysis (^51^Cr release) was performed with a gamma counter (Wizard 1470 gamma counter Pharmacia – LKB, Turku, Finland) using the following equation: [(Exp – Sp)/(Max – Sp) × 100]. All the results are presented at E:T = 50:1 ratio.

### Hematological Analysis of Leukocyte, Lymphocyte, Granulocyte and Monocyte Numbers

Total white blood cell (WBC) was counted using a Neubayer’s hemocytometer. The percentage of lymphocytes, granulocytes and monocytes was determined microscopically (under oil-immersion) on the whole blood smears after staining with Giemsa and May-Grünwald method (Hematology slide stainer 7120, Vescor, USA). The absolute number of blood lymphocytes, granulocytes and monocytes was calculated as absolute leukocyte number multiplied by the percentage of the respective leukocyte subpopulation.

### Cytometric Analysis of Lymphocyte Population and PBMC Apoptosis

#### Immunophenotypic Analysis of Lymphocyte Populations

The immunophenotypic analysis of peripheral blood lymphocytes was performed by staining PBMC with surface molecules with two panels. In panel 1, the general lymphocyte immunophenotyping (T, B and NK) was performed. The following three-color combination of fluorescent monoclonal antibodies and staining reagents cocktails were used: fluorescein isothiocyanate (FITC) conjugated anti-mouse CD3 (Clone: 1F4), phycoerythrin (PE) covalently linked to cyanine 7 conjugated anti-mouse CD45RA (Clone: OX-38), allophycocyanine (APC) conjugated anti-mouse CD161a (Clone: 10/78). The second panel, analyzed T lymphocytes subpopulations using the following three-color combination of fluorescent monoclonal antibodies and staining reagents FITC conjugated cocktails: anti-mouse CD3 (Clone:1F4); PE7 conjugated anti-mouse CD4 (Clone: OX-33), APC conjugated anti-mouse CD8 (Clone: OX-8). All the reagents were purchased from Beckman Coulter.

The sample preparation and analysis were carried out in accordance with the procedure described previously (Listowska et al. 2015). For each rat, two samples of PBMC adjusted to 1 × 10^6^ cells/ml in complete medium (RPMI, Sigma-Aldrich, Poland) were used for analysis. PBMC viability was evaluated using trypan blue dye (Trypan Blue solution, Sigma-Aldrich, Poland) and viable cells were counted under microscopy. Minimum cell viability was 80%. Twenty five microliters of such a cell suspension was added to 25 μl of an antibody cocktail specific for the desired cell populations (IOTest CD3-FITC/CD45RA-PC7/CD161a-APC or CD3-FITC/CD4-PC7/CD8-APC; Beckman Coulter, USA) according to the manufacturer’s instructions. Then, the samples were mixed and incubated at room temperature for 20 min in darkness. After incubation, 25 μl of Fixative Solution (Beckman Coulter, USA) and 700 μl of PBS were added to each sample. The samples were protected from light and stored at 4 °C until flow cytometry was performed with a Cytomics FC 500 flow cytometer (Beckman Coulter, USA) and MXP Software.

**Fig. 2:**
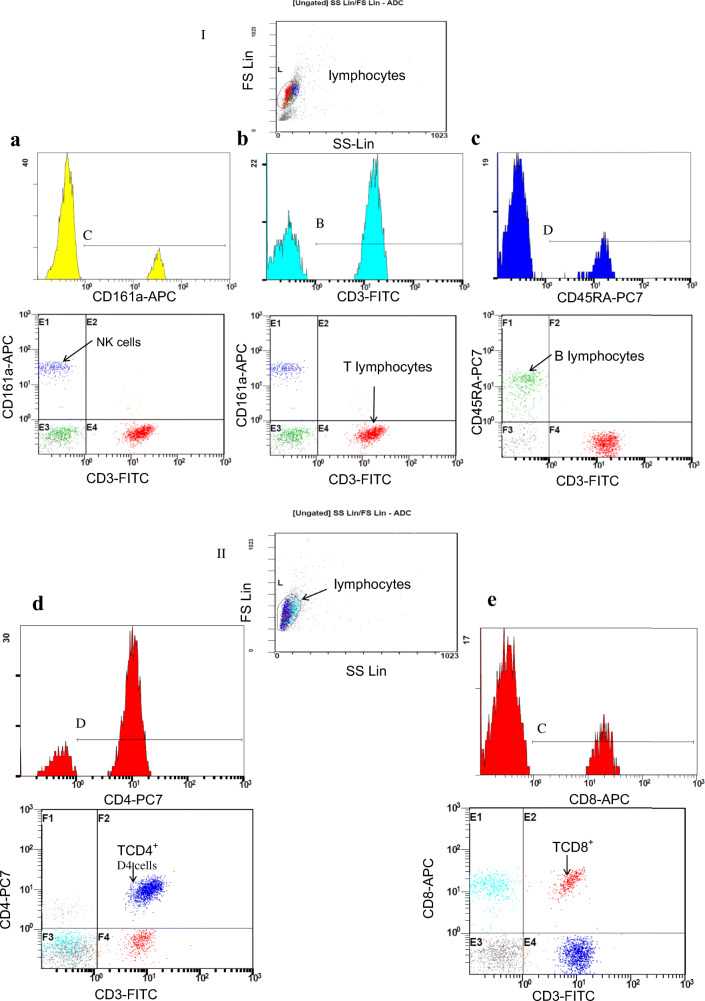
Histograms, gating strategy and representative flow cytometric analysis of general lymphocytes populations (I) and subsets of T lymphocytes (II). FS vs. SS graph, circle highlights the lymphocyte population. Identification of different lymphocytes populations in PBMC based on CD3, CD161a and CD45RA expression: (**a**). NK cells (CD3^−^CD161a^+^); (**b**). T lymphocytes (CD3^+^ CD161a^−^); (**c)**. B lymphocytes (CD3^−^CD45RA^+^) and CD3, CD4, CD8 expression: (**d)**. T CD4 (CD3^+^CD4^+^CD8^−^); E. T CD8 (CD3^+^CD4^−^CD8^+^) are shown

Samples were accepted if at least 1000 cells were counted. The lymphocytes were initially identified based on forward-scatter (FS) and side-scatter (SS) characteristics (Fig. 2). Then, the main lymphocyte populations were gated based on CD3, CD161a and CD45RA expression. Figure 2A shows the analysis for gating on the CD3^−^CD161a^+^ NK cells. Figure 2B shows the analysis for gating on the expression level of CD3^+^CD161a^−^ T lymphocytes. Figure 2C shows the analysis for gating on CD45RA^+^ CD161a^−^ B lymphocytes. Among CD3^+^ T cells, we further identify the CD8^+^ and CD4^+^ subtypes. We analyzed phenotypes of T lymphocytes gating TCD3^+^CD4^+^CD8^−^ (Fig. 2D) and TCD3^+^CD4^−^CD8^+^ (Fig. 2E) lymphocytes, respectively. The absolute numbers of cells in each lymphocyte population (T, B or NK) or T subpopulation were calculated according to the following formula:

$$ X\ \left[ No{}^{\circ}\frac{}{\mu l}\right]=\frac{Y\ \left[ No{}^{\circ}\frac{}{\mu l}\right] xZ\%}{100\%} $$

Explanation:

X – cell number in a lymphocyte population (T, B, or NK) or in a T lymphocyte subpopulation (TCD4^+^ or TCD8^+^).

Y - total number of all lymphocytes in a sample or total number of T lymphocytes in a sample.

Z – percentage of T, B or NK or (TCD4^+^ or TCD8^+^) (count from flow cytometry).

#### Percentage of PBMC Apoptosis

The apoptosis were detected by a previously used method (Listowska et al. 2015) that employs annexin-V to bind phosphatidylserine translocated to the cell surface during early apoptosis (Annexin V – FITC Kit, Beckman Coulter, USA) and propidium iodide (PJ) staining. Cell preparations were analyzed by a Cytomics FC 500 flow cytometer (Beckman Coulter, USA) and MXP Software. Cells considered to be undergoing apoptosis were those positive for annexin – V, but negative for PJ; cells already dead or in the late stages of apoptosis were positive for both annexin – V and PJ, and were not included.

### Histological Control of 6-OHDA Lesion and Electrode Placement

One hour after the end of DBS-STN or sham stimulation, rats were euthanized with Morbital (2 ml/kg) and transcardially perfused (via the left ventricle) with 200 ml of 0.9% saline followed by 200 ml of 4% paraformaldehyde in 0.1 M phosphate-buffered saline (PBS).

#### Tissue Preparation

The brains were removed quickly, postfixed and then cryoprotected in a 30% sucrose solution in PBS and then frozen, and kept at −70 °C until cryostat sectioning (CM 1850, Leica Biosystems, Germany). Frontal 30-μm-thick tissue sections were cut at −20 °C, at the level of the SNpc (between ventral tegmental area and STN) based on the stereotaxic atlas of Paxinos and Watson (2007).

#### Immunohistochemistry for TH-Expression

To determine the loss of dopaminergic neurons in the SNpc, we used immunohistochemical staining of tyrosine hydroxylase (TH) as previously described (for details see: Jerzemowska et al. 2012). Briefly, prior to all the immunohistochemical stages, the sections were rinsed several times in PBS, then incubated in 0.3% hydrogen peroxide in PBS for 10 min at room temperature and then blocked for 45 min with a solution of 1% Bovine Serum Albumin (BSA) (BioChemika, Fluka) and 0.3% Triton X-100 in PBS at room temperature for the effective reduction of nonspecific binding. Next, the sections were incubated with a polyclonal rabbit anti-TH antibody (Chemicon, Millipore, USA) at a dilution of 1:1500 (diluted in PBS containing 0.3% TritonX-100 and 3% Normal Goat Serum (NGS, Sigma-Aldrich, Poland) at 4 °C for 3 days. For light visualization, after a 30-min incubation with biotinylated goat anti-rabbit IgG (a dilution of 1:200; Vector, USA) in PBS containing 0.02% sodium azide and 0.3% Triton X-100 at room temperature, the sections were rinsed with PBS + Triton and incubated with avidin–biotin peroxidase complex (ABC) (a dilution of 1:100 in PBS; Vector Elite Kit, USA) for 1 h at room temperature. Washed in PBS, the sections were incubated in 40 ml Tris buffer (pH 7.6) (BioChemika, Fluka) containing 30 mg of diaminobenzidine tetrahydrochloride (DAB) (Sigma-Aldrich, Poland). After a few minutes, the sections were incubated with 30% hydrogen peroxide (H_2_O_2_) (solution 90 μl H_2_O_2_/10 ml PBS, Eurochem BGD, Tarnow, Poland), and allowed to react for 15–20 min. The reaction was controlled and stopped in Tris buffer when the TH-immunoreactive cells turned brown. The tissue sections were placed on slides, air dried and after dehydration with ethanol, mounted with DPX (DPX Mountant for histology, Sigma-Aldrich, Poland).

#### Microscopic Analysis

TH^+^ cell bodies were counted in three sections of the SNpc per region (−4.80 mm,  − 5.20 mm, − 6.04 mm) for each rat. Then, the mean value of TH^+^ neurons from each animal was expressed as a percentage cell loss of the lesioned side compared to non-lesioned side. Only animals with more than 90% depletion of dopaminergic neurons in the SNpc when compared to the non-lesioned side, were taken to statistical analysis (Fulceri et al. 2006).

To determine the location of the stimulating electrode we used the Nissl staining. The selected sections were placed on slides, stained with Cresyl violet (Sigma-Aldrich, Poland), dehydrated and finally mounted with DPX (Sigma-Aldrich, Poland). Animals showing a misplaced electrode were not included in the experimental groups presented above.

### Statistical Analysis

All statistical analyses were performed using IBM SPSS Statistics 21.0 and the level of significance was set at *p* ≤ 0.05. The statistical evaluation of the mean TH^+^ cells in the SNpc was assessed using two-way ANOVA with factors: experimental group (SHAM, DBS-6OHDA and 6OHDA) and laterality (right and left hemispheres in each experimental group), the differences in the means were further analyzed with Tukey’s HSD post hoc. The immune and endocrine parameters were compared using one-way ANOVAs and Tukey’s HSD post-hoc test. All data were expressed as the mean ± SEM.

## Results

### Histological Verification

#### TH Immunohistochemistry Confirmed Lesions in the SNpc

**Fig. 3:**
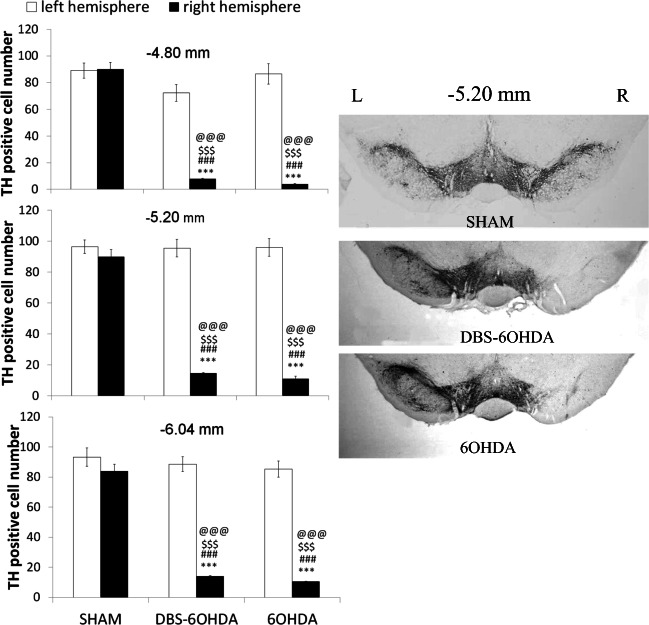
The number of tyrosine hydroxylase positive cells (TH^+^ cells) in the left (L) and right (R) brain hemisphere at the bregma level: −4,80 mm, −5,20 mm −6,04 mm in the deep brain subthalamic nucleus stimulated rats with PD model (DBS-6OHDA group, *n* = 14), non-stimulated rats with PD model (6OHDA group, *n* = 9), control (SHAM group, n = 14) group and representative photomicrographs of brain coronal sections (−5.20 mm) for rats in each group. Explanations: ***p ≤ 0.001 – difference vs. left hemisphere of DBS-6OHDA group; ###p ≤ 0.001 difference vs. left hemisphere of 6OHDA group, $$$p ≤ 0.001 difference vs. left hemisphere of SHAM group; @@@p ≤ 0.001 difference vs. right hemisphere of SHAM group; Tukey’s HSD post hoc after two-way ANOVA

Intranigral administration of 6-OHDA at the right brain hemisphere induced a large reduction of TH-immunoreactive neurons in the SNpc, as demonstrated by immunohistochemistry performed 26 days after neurotoxin injection (Fig. 3). Quantification of TH+ neurons revealed a significant reduction in the number of TH-positive body cells in the right SNpc of both DBS-6OHDA and 6OHDA animals. Two-way ANOVA for the number of TH positive neurons in the SNpc revealed effects of the experimental group (at 4.80: F_(1,222)_ = 63.38; *p* ≤ 0.001; at 5.20: F_(1,222)_ = 250.50, p ≤ 0.001; at 6.04: F_(1,222)_ = 248.96, p ≤ 0.001) and laterality (at 4.80: F_(4,222)_ = 59.61, p ≤ 0.001; at 5.20: F_(4,222)_ = 115.64, p ≤ 0.001; at 6.04: F_(4,222)_ = 98.30, p ≤ 0.001). Tukey’s HSD post hoc test revealed that the number of TH^+^ neurons was reduced in DBS-6OHDA group in the right SNpc relative to left SNpc, in both hemispheres of SHAM rats and in the left hemisphere of 6OHDA animals, at all analyzed Bregma levels (p ≤ 0.001, respectively, Fig. 3). The same effects were observed in 6-OHDA animals (Fig. 3).

#### Nissl Staining

**Fig. 4:**
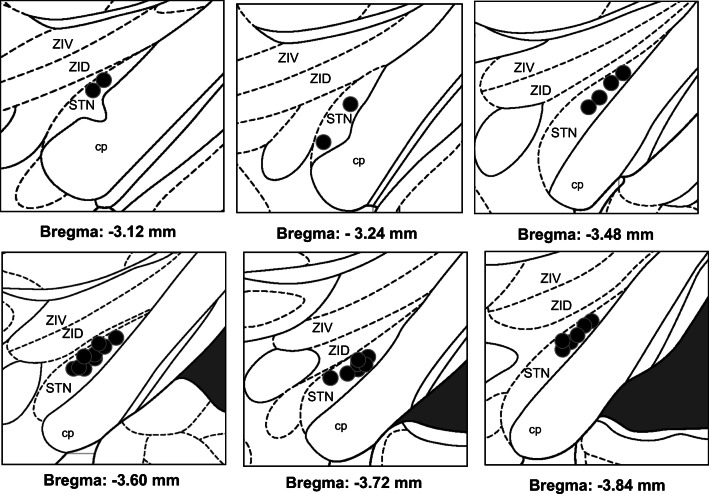
Location of electrodes tips (black dots) superimposed on plates adapted from the atlas by Paxinos and Watson (2007) in all subthalamic deep brain stimulated rats (DBS group, *n* = 12 and DBS-6OHDA group, n = 14). Explanations: STN, subthalamic nucleus; cp, cerebral peduncle; ZID, zona incerta pars dorsalis; ZIV, zona incerta pars ventralis

The stimulating electrode placement within the STN was confirmed with Nissl’s staining. In both DBS-STN stimulated groups, the electrode tips were placed at the level of 3.12 to 3.84 posterior to bregma (Fig. 4).

### Corticosterone and Cytokine Plasma Level

**Fig. 5:**
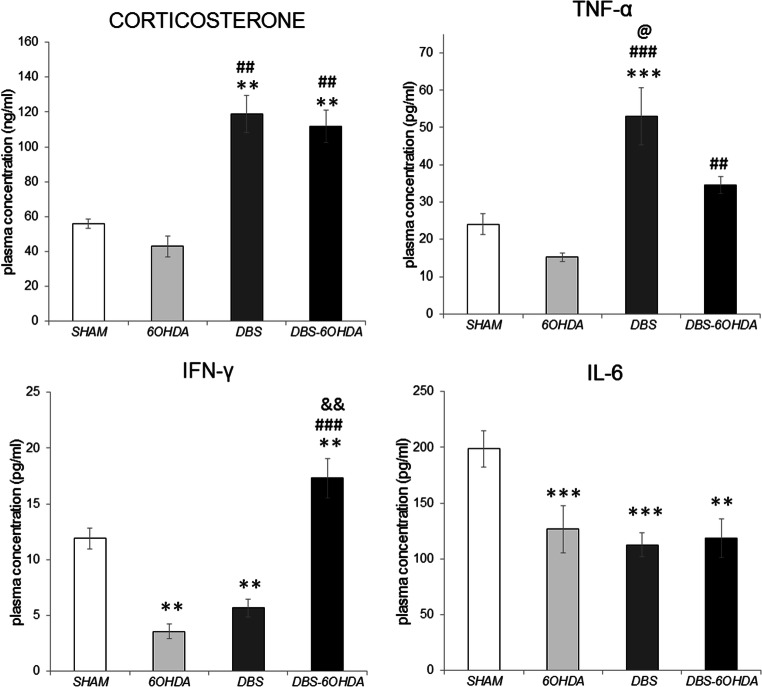
Plasma corticosterone and cytokine: tumor necrosis alfa (TNF-α), interferon gamma (IFN-γ) and interleukin 6 (IL-6) concentrations in subthalamic deep brain stimulated rats without nigral dopamine depletion (DBS group, n = 12), subthalamic deep brain stimulated rats with PD model (DBS-6OHDA group, n = 14), non-stimulated rats with PD model (6OHDA group, n = 9) and control (SHAM group, n = 14). Explanations: ***p ≤ 0.001; **p ≤ 0.01 – difference vs. SHAM group; ###p ≤ 0.001, ##p ≤ 0.01 – difference vs. 6OHDA group; &&p ≤ 0.01 difference vs. DBS group, @*p* ≤ 0.05 – difference vs. DBS-6OHDA group; Tukey post hoc after one-way ANOVA

Biochemical analysis of the level of CORT in blood samples were performed by radioimmunoassay method (^125^I RIA). One-way ANOVA showed a significant difference in plasma CORT concentrations (F (Benabid et al. 2009; Reale et al. 2009)_=_4.45, *p* ≤ 0.01) between groups (Fig. 5). The plasma CORT concentration in non-stimulated rats with PD model was lower than in both DBS-STN stimulated groups (DBS: *p* ≤ 0.01 and DBS-6OHDA: p ≤ 0.01, Tukey’s HSD, Fig. 5), but not than in SHAM. Post hoc tests also confirmed that in both stimulated groups (DBS, DBS-6OHDA) the levels of plasma CORT concentrations were elevated in comparison with SHAM (*p* ≤ 0.01 and p ≤ 0.01, Fig. 5).

Plasma cytokine concentrations were measured by ELISA. The statistical analysis showed that the TNF-α concentration in the stimulated groups, DBS and DBS-6OHDA (Fig. 5), was significantly elevated (F (Benabid et al. 2009; Reale et al. 2009)= 14.344, *p* ≤ 0.001) in comparison with non-stimulated rats with PD model (p ≤ 0.001 and p ≤ 0.01, respectively; Tukey’s HSD). Only animals in DBS group displayed an elevated level of plasma TNF-α (*p* ≤ 0.001; Tukey’s HSD; Fig. 5) in comparison with the SHAM group. The IFN-γ and IL-6 concentrations were down-regulated in 6OHDA (*p* ≤ 0.001 and p ≤ 0.001; Tukey’s HSD; Fig. 5) groups compared with the SHAM group. Plasma IFN-γ concentration was down-regulated in the DBS (*p* ≤ 0.01; Tukey’s HSD; Fig. 5) group compared with the SHAM group, while up-regulated in DBS-6OHDA (*p* ≤ 0.001; Tukey’s HSD; Fig. 5) animals (F (Benabid et al. 2009; Reale et al. 2009)=23.183). In addition, DBS-STN stimulated animals with PD model had higher level of plasma IFN-γ - than animals in both DBS and 6OHDA groups (*p* ≤ 0.01 and *p* ≤ 0.001; Tukey’s HSD; Fig. 5). In both stimulated groups (DBS, DBS-6OHDA, F (Benabid et al. 2009; Reale et al. 2009)=9498, p ≤ 0.001), IL-6 levels were markedly decreased compared with the SHAM group (p ≤ 0.001 and p ≤ 0.001; Tukey’s HSD; Fig. 5).

### Blood White Cells Absolute Number

**Fig. 6:**
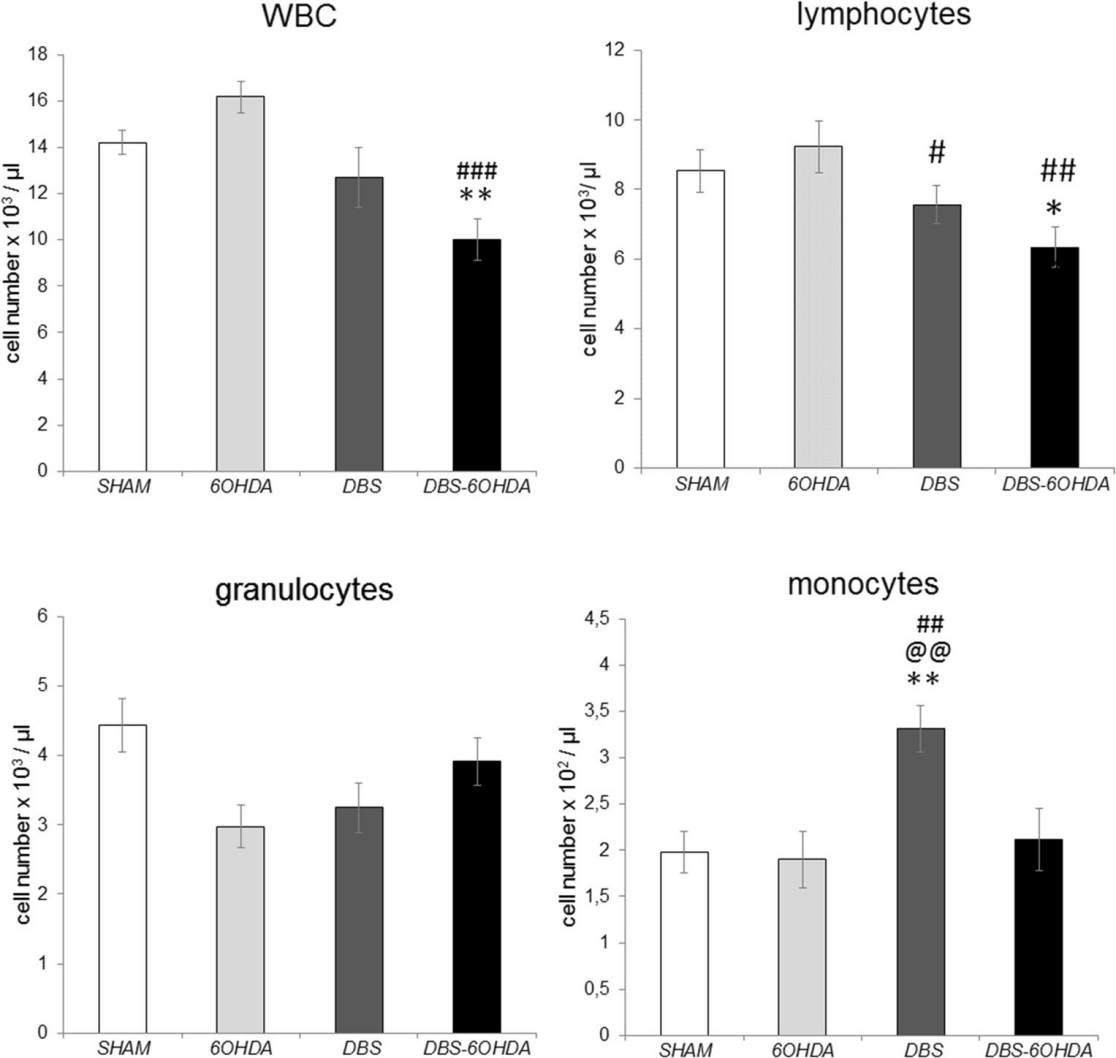
Total white blood cell (WBC), lymphocyte, granulocyte and monocyte number in subthalamic deep brain stimulated rats (DBS group, n = 12), subthalamic deep brain stimulated rats with PD model (DBS-6OHDA group, n = 14), non-stimulated rats with PD model (6OHDA group, n = 9) and control (SHAM group, n = 12). Explanations: **p ≤ 0.01 – difference vs. SHAM group; ###p ≤ 0.001, ##p ≤ 0.01 – difference vs. 6OHDA group; @@ p ≤ 0.01 – difference vs. DBS-6OHDA group; Tukey post hoc after one-way ANOVA

**Fig. 7:**
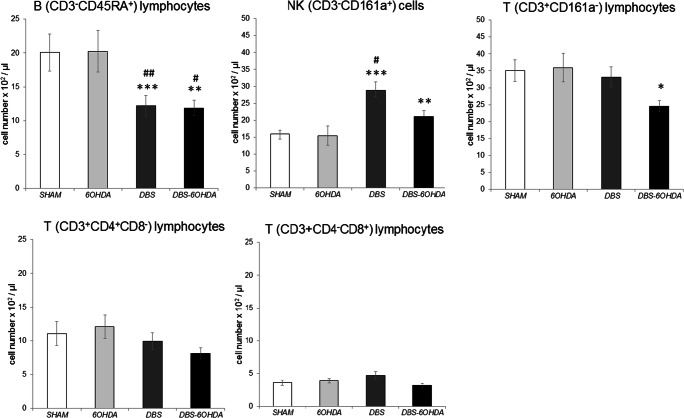
The absolute number of T (CD3^+^ CD161a^−^), B (CD3^−^CD45RA^+^), NK (CD3^−^CD161a^+^) cells and TCD4^+^ (CD3^+^CD4^+^CD8^−^) or TCD8^+^ (CD3^+^CD4^−^CD8^+^) subpopulation in peripheral blood of subthalamic deep brain stimulated rats without nigral dopamine depletion (DBS group, n = 12), subthalamic deep brain stimulated rats with PD model (DBS-6OHDA group, n = 14), non-stimulated rats with PD model (6OHDA group, n = 9) and control (SHAM group, n = 14). Explanations: ***p ≤ 0.001, **p ≤ 0.01, *p ≤ 0.05 – difference vs. SHAM group; ##p ≤ 0.01; #p ≤ 0.05 – difference vs. 6OHDA group; Tukey post hoc after one-way ANOVA

WBC were counted using a hemocytometer (Fig. 6) and also using flow cytometry for selected surface markers (Fig. 7). There were significant differences between the groups in the total WBC number (F (Benabid et al. 2009; Reale et al. 2009)=7.514, p ≤ 0.001, Fig. 6). Significant differences between groups were observed also in absolute numbers of lymphocytes (F (Benabid et al. 2009; Reale et al. 2009)=5.552, *p* ≤ 0.01), monocytes (F (Benabid et al. 2009; Reale et al. 2009)=7.766, p ≤ 0.001), T (CD3^+^CD161a^−^) lymphocytes (F (Benabid et al. 2009; Reale et al. 2009)=3.314, *p* ≤ 0.05), B (CD3^−^CD45RA^+^) lymphocytes (F (Benabid et al. 2009; Reale et al. 2009)=8.313, p ≤ 0.001) and NK (CD3^−^CD161a^+^) cells (F (Benabid et al. 2009; Reale et al. 2009)=8.369, p ≤ 0.001). As shown Fig. 6, the absolute number of lymphocytes was decreased in both stimulated groups (DBS and DBS-6OHDA) in comparison with 6-OHDA group (*p* ≤ 0.05 and *p* ≤ 0.01; Tukey’s HSD). The stimulation effects in DBS and DBS-6OHDA groups were observed also in absolute numbers of B (CD3^−^CD45RA^+^) lymphocytes (p ≤ 0.01 and *p* ≤ 0.001; Fig. 7) and NK (CD3^−^CD161a^+^) cells (*p* ≤ 0.001 and *p* ≤ 0.01; Fig. 7) in comparison with SHAM group. In addition, post hock tests showed significant differences in the absolute numbers of WBC (p ≤ 0.01; Fig. 6), lymphocytes (p ≤ 0.05; Fig. 6) and T (CD3^+^CD161a^−^) cells (p ≤ 0.05; Fig. 7) between the DBS-6OHDA and SHAM group. The monocytes absolute number were higher only in the DBS-stimulated animals without nigral dopamine depletion in comparison with SHAM group (p ≤ 0.01; Fig. 6).

### Lymphocytes Percentage in PMBC

Flow cytometric immunophenotyping was performed to analysis lymphocytes population. The data from this analysis are presented in Table 1. Based on the one-way ANOVA, there were stimulation effect on B (CD3^−^CD45RA^+^) lymphocytes (F (Benabid et al. 2009; Reale et al. 2009)=4.883, *p* ≤ 0.01) and NK (CD3^−^CD161a^+^) cell (F (Benabid et al. 2009; Reale et al. 2009)=5.242, *p* ≤ 0.01) percentage. The both DBS-STN groups (DBS and DBS-6OHDA) had a lower percentage of B (CD3^−^CD45RA^+^) lymphocytes than SHAM group (*p* ≤ 0.01 and *p* ≤ 0.05). In contrast, the percentages of NK (CD3^−^CD161a^+^) cells were higher in DBS and DBS-6OHDA groups than in SHAM group (p ≤ 0.01 and p ≤ 0.05). In addition, one-way ANOVA showed significant effects of nigrostriatal lesion and DBS-STN stimulation on TCD8^+^ (TCD3^+^CD4^−^CD8^+^) (F (Benabid et al. 2009; Reale et al. 2009)=25.218, *p* ≤ 0.001) percentage. The counts of TCD8^+^ in 6OHDA and DBS-6OHDA groups were higher than in SHAM group (p ≤ 0.001 and p ≤ 0.001), while in DBS and DBS-6OHDA groups lower than in 6OHDA group (p ≤ 0.001 and p ≤ 0.01). The opposite trend was noted for TCD4^+^ (TCD3^+^CD4^+^CD8^−^) lymphocytes percentage, but there were no significant difference between groups.

**Table 1:** The percentage of T (CD3^+^CD161a^−^), B (CD3^−^CD45RA^+^), NK (CD3^−^CD161a^+^) cells and TCD4^+^ (CD3^+^CD4^+^CD8^−^) or TCD8^+^ (CD3^+^CD4^−^CD8^+^) subpopulation in peripheral blood mononuclear cells (PBMC) of subthalamic deep brain stimulated rats without nigral dopamine depletion (DBS group, n = 12), subthalamic deep brain stimulated rats with PD model (DBS-6OHDA group, n = 14), non-stimulated rats with PD model (6OHDA group, n = 9) and control (SHAM group, n = 14)

	PBMC PERCENTAGE ± SD				
	T (CD3^+^CD161a^−^)	B (CD3^−^CD45RA^+^)	NK (CD3^−^CD161a^+^)	T (CD3^+^CD4^+^CD8^−^)	T (CD3^+^CD4^−^CD8^+^)
SHAM	40.3 ± 4.1	22,8 ± 2.3	18.4 ± 1.1	72.8 ± 6.7	27.2 ± 4.3
6OHDA	44.9 ± 2.9	21,3 ± 1.2	21.2 ± 1.8	65.2 ± 5.1	34.8 ± 2.2 ***
DBS	40.3 ± 2.4	16,3 ± 1.0 **	28.6 ± 2.8 **	74.9 ± 6.1	25.1 ± 2.7; ###
DBS-6OHDA	42.8 ± 4.3	16,9 ± 1.3 *	25.9 ± 2.5 *	70.5 ± 3.9	29.5 ± 1.7 ***; ##

Explanations:

***p ≤ 0.001, **p ≤ 0.01, *p ≤ 0.05 – difference vs. SHAM group; ##p ≤ 0.01; #p ≤ 0.05 – difference vs. 6OHDA group; Tukey post hoc after one-way ANOVA

### PBMC Apoptosis and NK Cell Cytotoxic Activity

**Fig. 8:**
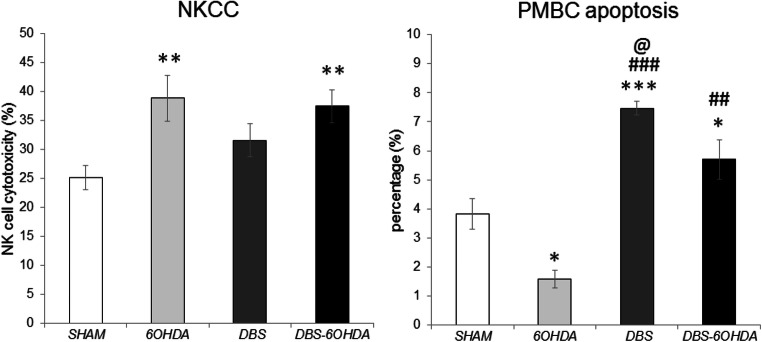
Blood natural killer cell cytotoxicity against YAC-1 target cells at effector: target (E:T) cell ratio of 50:1 and peripheral blood mononuclear cells (PBMC) apoptosis in subthalamic deep brain stimulated rats without nigral dopamine depletion (DBS group, n = 12), subthalamic deep brain stimulated rats with PD model (DBS-6OHDA group, n = 14), non-stimulated rats with PD model (6OHDA group, n = 9) and control (SHAM group, n = 12). Explanations: **p ≤ 0.01, *p ≤ 0.05 – difference vs. SHAM group; ###p ≤ 0.001; ##p ≤ 0.01– difference vs. 6OHDA group; @ p ≤ 0.05 – difference vs. DBS-6OHDA group; Tukey post hoc after one-way ANOVA

The PBMC apoptosis were analyzed by flow cytometry using FITC-annexin V and PI double staining. The percentage of apoptotic cells detected in 6-OHDA group was lower than in SHAM animals (F (Benabid et al. 2009; Reale et al. 2009)=17.505, p ≤ 0.001; p ≤ 0.05, Tukey’s HSD; Fig. 8). The opposite effects were observed in DBS and DBS-6OHDA groups compared with SHAM group (p ≤ 0.001 and p ≤ 0.05; Tukey’s HSD; Fig. 8). In addition, in both stimulated groups (DBS, DBS-6OHDA), the mean percentages of apoptotic cells in PBMC were higher than in 6-OHDA rats (p ≤ 0.001; p ≤ 0.01; Tukey’s HSD; Fig. 8).

Using a ^51^Cr release assay, we demonstrated that the NKCC was higher in both dopamine depleted groups (6-OHDA and DBS-6OHDA) in comparison to SHAM (F (Benabid et al. 2009; Reale et al. 2009)=5.632, p ≤ 0.01; p ≤ 0.01 and p ≤ 0.01; Tukey’s HSD; Fig. 8).

## Discussion

DBS-STN ameliorates the major motor symptoms of PD (Benabid et al. 2009; Faggiani and Benazzouz 2017) and reverses certain electrophysiological and metabolic consequences of dopamine depletion in rats (Winter et al. 2008; He et al. 2014). Spectacular effects of DBS-STN in PD patients may occur with neuropsychiatric complications (Nassery et al. 2016). In order to study the mechanism involved in side effects of DBS-STN, we investigated several markers of the peripheral inflammatory response after DBS-STN applied in normal and PD rats, and compare the obtained results with the rat model of PD. The present study demonstrates that DBS-STN influences peripheral blood immune responses, probably via the corticosterone-dependent mechanism. We observed that, after DBS-STN in rats, plasma CORT level was elevated. The endocrine effect was associated with a decrease in the total white cells and lymphocyte numbers, especially B and T lymphocytes in DBS-STN stimulated rats with PD model. In contrast, DBS-STN applied in rats without PD model did not influence T lymphocytes number and decreased the number of B lymphocytes. Concomitantly with CORT and TNF-α elevated levels, a higher count of apoptotic cells in blood was observed after DBS-STN. Surprisingly, the number and percentage of NK cells increased after DBS-STN, while the number of monocytes increased only after DBS-STN applied in rats without PD model. It is worth underlining, that in both dopamine depleted groups (6OHDA and DBS-6OHDA) elevated levels of NKCC were observed. It seems that in DBS-6OHDA rats, this functional activation of NK cells occur with an increase in the percentage and number of NK cells and IFN-γ level. On the contrary, IFN-γ levels were down regulated in 6OHDA rats, but the percentage of T cytotoxic cells (TCD3^+^TCD8^+^) were higher than in DBS-STN stimulated rats. As far as we know, there are no studies on DBS-STN effects on the immune response in rats with PD model, so the present study is pioneering.

It seems that DBS-STN can influence the immune system through hormonal, neurotransmitter and cytokine pathways that are claimed to be involved in neuro-immune interactions. Dopamine plays an important role in the regulation of immune responses, including NK cell activity and number, leukocyte subpopulation distribution, cytokine production and modulation of cytokines for B cell development (Dhabhar et al. 2012). There is accumulating evidence that DBS-STN increases striatal dopamine efflux and metabolism in rats (Salin et al. 2002; Meissner et al. 2002; Lee et al. 2006; He et al. 2014) and activates the autonomic system in PD patients (Furgała et al. 2015). However, the present design of the experiment does not allow us to differentiate whether immunomodulative effect of DBS-STN is mediated by direct modulation of the STN or by the current spreading to adjacent structures. For instance, the STN is directly adjacent to the VTA (Haegelen et al. 2009). Recent study shows, that DBS-STN applied in rats, increased neuronal activity as measured by c-Fos expression in the nucleus accumbens, the part of the mesolimbic system (Hachem-Delaunay et al. 2015). Thus, DBS-STN might relieve the VTA dopaminergic neurons from their inhibitory tone by modulating the excitatory driver on the VTA neurons. On the contrary, the blockade of dopamine-releasing neurons in the VTA resulted in depression-like symptoms, such as decline of motivation to explore new settings. Alternatively, by stimulating dopaminergic neurons in the VTA, depressive behaviors are restored (Tye et al. 2013). Thus, the neuronal activation of the VTA following DBS-STN may be related to the development of side effects observed in PD patients. In our study, we previously observed immunostimulative effects on NK cell number and activity after chronic electrical stimulation of the VTA (Wrona et al. 2004) and BST (Myślińska et al. 2012) in rats. That immunomodulatory effect of DBS-STN reported in this work may by related with influence of DBS-STN on the mesolimbic system and CORT secretion. Indeed, in our study, DBS-STN may hypothetically act on the hypothalamus and increase dopamine release, which activates the HPA-axis with a subsequent increase in the production of such an immunomodulatory hormone as CORT. The elevated level of CORT in plasma may lead to peripheral suppressive effects on the number of lymphocytes, especially B and T cells. In addition, the lymphocyte apoptosis level and the production of such pro-apoptotic, Th1 type cytokines as TNF-α and IFN-γ were affected. This suggests two different mechanisms of blood lymphocyte number changes. First, recirculation as the main and most probable cause of monocyte and NK cell number increase and second, leukocyte migration or apoptosis of blood B and T cells. CORT induces immune cells traffic out of the blood possibly to tissue surveillance pathways, lymphoid tissues, and sites of ongoing or de novo immune activation and in some cases, stimulated NK cells activity in peripheral blood (Dhabhar et al. 2012). In our study elevated CORT concentration in plasma was detected one hour after the end of DBS-STN. So changes in blood lymphocyte number after DBS-STN, may be due to the activation of the immune system similar to that observed after stress.

Leukocyte migration is a crucial process in both homeostatic and inflammatory conditions. Recruitment of immune cells into the central nervous system (CNS) has also been reported in many infectious diseases and neurodegenerative disorders (Wilson et al. 2010). Theodore and Maragos (2015) showed that in mice, 6-OHDA administration induced an intense IgG deposition in the SN as well as increased infiltration of both T and B lymphocytes into the injected side of the midbrain. The relevance of the infiltrating peripheral monocytes in the degenerative event in PD has been confirmed in rodent PD models of dopaminergic degeneration (Tansey and Romero-Ramas 2019). The CD163^+^ anti-inflammatory targeted modification of monocytes in the 6-OHDA rat model using dexamethasone (Tentillier et al. 2016) or the deletion of the CCR2 (thus avoiding macrophage recruitment) in the viral-vector-α-syn PD mouse model (Harms et al. 2018) both resulted in dopaminergic neuronal protection in the SN. In addition, Ip et al. (2015) found that the immune system plays a crucial role in modulating neurodegeneration in the 6-OHDA mouse model of PD by significant elevation of TCD8^+^ lymphocyte population within the striatum after toxin injection and a protective role of lymphocyte invasion and neuroinflammation in immune competent mice. Indeed, in our study we found that percentage of TCD8^+^ lymphocytes in peripheral blood of 6OHDA rats was higher than in both DBS-STN stimulated groups. On the other hand, a study of Fu et al. (2017) demonstrated that the administration of 6-OHDA in mice increased the expression of endothelial adhesion molecules, such as intercellular adhesion molecule 1 (ICAM-1), vascular cell adhesion molecule 1 (VCAM-1), and E-selectin. This data confirmed that, after 6-OHDA administration, the blood-brain barrier (BBB) is more permeable. In our study, the number of T and B lymphocytes in DBS-STN rats with PD model decreased in peripheral blood. This effect may be related to the migration of lymphocytes to brain regions activated during inflammation caused by 6-OHDA injection. In addition, several lines of evidence confirm that DBS-STN applied in PD patients increases the global (whole-brain) cerebral blood flow (Sidtis et al. 2012; Mubeen et al. 2018). Thus, DBS-STN applied in rats with PD can facilitate migration of T and B lymphocytes trough more permeable BBB. Unfortunately, in the present study, we did not analyze the markers of neuroinflammation and leukocyte trafficking in brain regions, so this mechanism must be confirmed by further experiments.

Studies that are dealing with potential changes in the number and/or function of NK cells in peripheral blood of PD patients are limited. In a study of Mihara et al. (2008) and Niwa et al. (2012), an elevated percentage of NK cells in PD patients with no changes in activity of NK cells in PD patients and healthy controls was confirmed. In addition, NKCC in PD patients was positively correlated with PD severity (Mihara et al. 2008). The recent study by Earls et al. (2020) demonstrated that in vitro, α-syn aggregates attenuated NK cell cytotoxicity in a dose-dependent manner. Importantly, systemic depletion of NK cells in the mice model of PD led to exacerbated synuclein pathology and motor deficits, suggesting a protective role of NK cells in Lewy body-related neurodegenerative diseases (Earls et al. 2020). Although it has been reported that 6-OHDA does not interact with α-synuclein and 6-OHDA does not produce or induce proteinaceous aggregates or Lewy-like inclusions like those seen in PD (Blandini et al. 2008; Walsh et al. 2011), 6-OHDA-induced activation of glial populations such glial cell markers as GFAP (anti-glial fibrillary acidic protein) for astrocytes (Walsh et al. 2011; Mulas et al. 2016), OX-42 for microglia (Marinova-Mutafchieva et al. 2009; Mulas et al. 2016) and neuronal TNF-α (Mulas et al. 2016). Such markers of neuroinflammation have been observed to occur from the second (Mulas et al. 2016) to the fourth (Walsh et al. 2011) week after the lesion. Moreover, Gasparotto et al. (2017) showed that the receptor for advanced glycation endproducts (RAGE) inhibition blocks all signaling cascades involved in neuroinflammation and dopaminergic denervation induced by 6-OHDA administration. In our study we found, that after 6-OHDA microinjection into the SNpc (in DBS-6OHDA and 6OHDA groups) the peripheral NKCC was elevated. This effect occurred with an increased number of blood NK cell in DBS-6OHDA group. A major implication of these observations would be that the NKCC changes were probably related to the neuroinflammation processes in the central nervous systems. The alterations of the physiology of NK cells observed in our study suggest that NK cells might be actively involved in PD progression, but not initiation.

Proinflammatory and neurotoxic cytokines and chemokines produced by activated microglia result in disruption of the BBB and attract lymphocytes (Kustrimovic et al. 2019). It has been documented that NK cells can be recruited to the CNS after several pathological conditions and their production of IFN-γ has been shown to be implicated in either neuroprotection or neurotoxicity (Poli et al. 2013). The activation of HPA-axis observed after DBS-STN leads to redistribution of NK cells to peripheral blood in both stimulated groups (DBS, DBS-6OHDA). Yet, only in DBS-STN stimulated rats with PD model, the increase in the number of NK cells was associated with elevated NKKC and IFN-γ plasma levels. That effect, as well as changes in the number of other lymphocyte populations, may be associated with increased blood flow during DBS-STN and the enhancement of BBB permeability due to the 6-OHDA microinjection.

The behavioral effects of DBS-STN in rats, which were observed in other studies (Creed et al. 2013; Hachem-Delaunay et al. 2015), support thesis that peripheral immunity changes may be related to the influence of stimulation on limbic associative regions. Unfortunately, the design of our study did not allow us to elucidate behavioral abnormalities after DBS-STN, but side effects of DBS-STN observed in Parkinsonian patients such as depression or anxiety are often associated with immune system functional changes. There is growing evidence to support the macrophage theory of depression (Beumer et al. 2012). In particular, tissue stress or malfunction, both in the brain and in the periphery, produce sustained inflammatory states, which may cause depression. Excessive release of proinflammatory mediators is responsible for alterations of neurotransmitter systems and the occurrence of depressive symptoms. In fact, in our study the elevated CORT plasma level and increased number of peripheral blood monocytes and NK cells after DBS-STN in normal rats were observed. In parallel with elevated plasma CORT level, such proinflammatory cytokine as TNF-α was elevated after DBS-STN in normal rats. However, DBS-STN applied in rats with PD model (DBS-6OHDA group) had no effect on monocytes number and TNF-α level. The inflammatory response develops after the recognition of disturbances, and proinflammatory mediators are secreted at the beginning of the immune reaction. Peripherally generated proinflammatory signals are transmitted into the CNS and induce sickness behavior – a complex of behavioral manifestations resembling a depressive state. Recent studies have demonstrated increased levels of pro-inflammatory cytokines (IL-6 and TNF-α) in Parkinsonian rat model (Gasparotto et al. 2017) and PD model associated with depression (Dallé et al. 2017). Surprisingly, in this study, the level of plasma TNF-α in rats with PD model did no differ from SHAM group. The reason of that effect may be related with the electrode implanted to the STN. After DBS electrode implantation, regional neuroinflammation in the rats was observed with concomitant memory impairment (Hirshler et al. 2010); in the macaque, reactive gliosis was seen after 3 months and 3 years (Griffith and Humphrey 2006); and in the human, reactive gliosis was observed in a postmortem study 12 years after implantation in an essential tremor patient with good tremor control (DiLorenzo et al. 2010). Recent study by Campos et al. (2020) showed that DBS activated astrocytes and prevented TNF-α-induced increase of monocyte chemoattractant protein-1 (MCP-1) and NF-κB activation in vitro. In the same study, 6-OHDA microinjection into the SN induced an increase in the number of GFAP-positive cells in the globus pallidus (GP) 13 days after PD model induction, but also influenced IFN-γ induction. In addition, 6-OHDA-induced IFN-γ was completely attenuated by DBS-STN (Campos et al. 2020). The above data suggests that in both stimulated groups, the peripheral immunity changes after DBS-STN could depend on the combined neuronal–glial interactions and influence on hormone balance.

One potential limitation of our study is DBS-STN duration. While in PD patients DBS-STN is applied chronically, in our study effects of 1 h DBS-STN stimulation were analyzed. Melon et al. (2015) showed that acute DBS-STN reversed increases in glutamate (Glu) and gamma aminobutyric acid (GABA) levels induced by dopamine lesion in the striatum in rats. Windels et al. (2000) shows that 1 h of DBS-STN in normal rats increases extracellular Glu level in the output nuclei of the STN, GP, and the substantia nigra pars reticulata (SNr), consistent with an increase in the activity of the STN neurons. DBS-STN also increases GABA levels in the SNr. On the contrary, the microdialysis studies by Windels et al. (2005) in PD rats showed that DBS-STN applied for 1 h did not affect extracellular Glu level in the SNr but doubled the level of GABA. In our study, we analyzed the effects of DBS-STN on immune and endocrine parameters and we speculate that these effects can be related to emotional dysfunction observed after therapy applied in PD patients. The ventral striatum is a main anatomical substrate for integration among functional networks between the basal ganglia and limbic regions. So changes at the level of neurotransmission in the striatum, GP and SNr seem to be crucial to link DBS-STN effects with emotional side effects. Obtained data can provide some clues toward understanding side effects observed after DBS-STN. To the best for our knowledge, our data is the first to suggest that the STN plays immunoregulatory role in several peripheral blood responses in rats. DBS-STN results in changes in the numbers of blood lymphocytes and their function which can be related to the influence of the stimulation on HPA-axis activity and endocrine-cytokine interactions. However, further studies are necessary to explain the mechanism of DBS-STN immunoregulatory effects and their clinical implication.
